# Effects of Interventions for the Prevention and Management of Maternal Anemia in the Advent of the COVID-19 Pandemic: Systematic Review and Meta-Analysis

**DOI:** 10.2196/57626

**Published:** 2025-10-06

**Authors:** John Kyalo Muthuka, Dianna Kageni Mbari-Fondo, Francis Muchiri Wambura, Kelly Oluoch, Japheth Mativo Nzioki, Everlyn Musangi Nyamai, Rosemary Nabaweesi

**Affiliations:** 1 Department of Community Health and Health Promotion, Faculty of Public Health, Kenya Medical Training College, Mbagathi Way, Kenyatta National Hospital, Nairobi, 30195-00100, Kenya, 254 724274843; 2 Epidemiology/Public Health Section, KEMRI Graduate School of Health Sciences, Kenya Medical Research Institute, Nairobi, Kenya; 3 Alberta Health Services, Edmonton, AB, Canada; 4 Kenya Medical Training College, Nairobi, Kenya; 5 School of Nursing, Andrews University, Berrien Springs, MI, United States; 6 School of Global Health, Meharry Medical College, Nashville, TN, United States

**Keywords:** maternal anemia, anemia in pregnancy, COVID-19, pregnancy complications, meta-analysis, maternal and child health, anemia prevention, reproductive health

## Abstract

**Background:**

The COVID-19 pandemic presented many unknowns for pregnant women, with anemia potentially worsening pregnancy outcomes due to multiple factors.

**Objective:**

This review aimed to determine the pooled effect of maternal anemia interventions and associated factors during the pandemic.

**Methods:**

Eligible studies were observational and included reproductive-age women receiving anemia-related interventions during the COVID-19 pandemic. Exclusion criteria comprised non-English publications, reviews, editorials, case reports, studies with insufficient data, sample sizes below 50, and those lacking DOIs. A systematic search of PubMed, Scopus, Embase, Web of Science, and Google Scholar identified articles published between December 2019 and August 2022. Risk of bias was evaluated using the Cochrane Risk of Bias 2 tool for randomized trials and the National Institutes of Health’s assessment tool for observational studies. Pooled rate ratios (RRs) with 95% CIs were calculated in Review Manager 5.4.1. Synthesis included subgroup analysis, meta-regression, and publication bias checks to assess intervention effectiveness.

**Results:**

This meta-analysis included 11 studies with 6129 pregnant women. Of these, 3591 (59%) were in the intervention group and 2538 (41%) were in the comparator group. Effects were recorded for 1921 (53.4%) women in the intervention group and 1350 (53.1%) in the comparator group. The cumulative impact ranged from 23% to 81%, averaging 56%. The initial analysis showed no significant effect on anemia prevention (RR 0.79, 95% CI 0.61‐1.02; *P*=.07), with high heterogeneity (*I*²=97%). Sensitivity analysis excluding 4 outlier studies improved the effect size to a significant level at 39% (RR 0.61, 95% CI 0.43‐0.87; *P*=.006). Subgroup analysis revealed substantial heterogeneity (*I*²=87.2%). Intravenous sucrose had a poor impact (RR 1.31, 95% CI 1.17‐1.47; *P*<.001), while medicinal or herbal interventions showed benefit (RR 0.81, 95% CI 0.73‐0.90; *P*=.006). Educational interventions yielded a 28% effect (RR 0.72), medicinal administration 19% (RR 0.81), iron supplementation 17% (RR 0.83), and intravenous ferric carboxylmaltose 15% (RR 0.85; *P*<.02). Additional sensitivity analysis confirmed a pooled positive effect of 17% (RR 0.83, 95% CI 0.79‐0.88; *P*<.001), with minimal heterogeneity (*I*²=0%). Regionally, effectiveness was highest in Africa (RR 0.84, 95% CI 0.79‐0.89; *P*<.001). Multicenter studies and those with 2020 data were predictive of better outcomes (RR 0.84 and RR 0.50, respectively). Despite initial heterogeneity and publication bias, interventions showed utility in mitigating maternal anemia in targeted subgroups and regions.

**Conclusions:**

Maternal anemia interventions during the COVID-19 pandemic showed modest, context-specific effectiveness, with declining impact from 2020 to 2022. Although high heterogeneity and study inconsistencies limited generalizability, significant benefits were observed particularly in African and multicenter studies. The pandemic exposed gaps in maternal health systems, emphasizing the need for tailored interventions, stronger data infrastructure, and resilient care strategies in future global crises.

## Introduction

Anemia is a condition where the number of red blood cells or the hemoglobin concentration within them is lower than normal. Maternal anemia refers to pregnant women having hemoglobin levels less than 12 g/dL [1-3]. Studies have found a correlation between the prevalence of anemia in women and the gross domestic product per capita. Projections suggest a 10% decline in global gross domestic product due to COVID-19, with findings indicating that the availability of nutritious foods, in particular, has been affected by COVID-19 measures [4].

Globally, the COVID-19 pandemic has had devastating effects on health care delivery systems for people of all ages, but pregnant women face particular challenges [5,6]. Reports show that the pandemic is making it increasingly challenging to provide adequate maternity care worldwide [5,7]. Even the movement of people seeking to access health care services has been restricted in many countries to prevent the spread of the virus. The pandemic has led to a complete stoppage of the import and export of many essential commodities among various countries, leading to a shortage of necessary items and affecting health care services badly, especially sexual and reproductive health care [8,9]. The population was advised not to go to hospitals unless strictly necessary; this advice seems to apply to all, including healthy pregnant women and even those with complications [5,10].

Before COVID-19, anemia prevention interventions focused on iron and folic acid supplementation, dietary modifications, and public health campaigns [11-13]. During the COVID-19 pandemic, these interventions adapted to include telemedicine, remote consultations, and increased community health worker involvement to address health care disruptions [14-17]. These measures aimed to ensure continued support for pregnant women [18,19]. Interventions to prevent anemia in pregnant women included iron and folic acid supplementation, dietary modifications, education and awareness programs, telemedicine and remote consultations, and community-based interventions [20,21]. The World Health Assembly set 6 targets to be accomplished by the year 2025. Among the targets is a 50% reduction of anemia in women of reproductive age through several strategies such as food fortification with iron, folic acid, and other micronutrients; the distribution of iron-containing supplements; and the control of infections and malaria [22].

There were many unknowns for pregnant women during the COVID-19 pandemic. Some issues may have gone unnoticed; however, conditions such as anemia could lead to worse pregnancy outcomes. Standard intervention efforts may have been compromised due to the COVID-19 pandemic, as was reported during prior pandemics, affecting the effect of health interventions in vulnerable populations [23].

The COVID-19 pandemic has had significant direct and indirect effects on pregnant women, newborns, young children, and adolescents. Directly, pregnant women infected with COVID-19 faced increased risks of preterm birth and stillbirth. However, the transmission of the virus from pregnant women to their newborns was found to be very low [10,24-26]. Indirectly, the pandemic led to reduced prenatal care visits, strained health care infrastructure, and increased maternal mental health issues such as anxiety and depression. Additionally, social and economic disruptions caused by the pandemic exacerbated domestic violence and financial instability, disproportionately affecting women and children [10].

These combined effects highlight the need for targeted interventions to support maternal and child health during and after the pandemic. The effects on pregnant women, newborn babies, young children, and adolescents are enormous and possibly translate to interventions meant to mitigate anemic conditions in pregnancy [6,7,9,10,27-29]. The objective of the review was to assess the cumulative impact of interventions for maternal anemia and related factors during the COVID-19 pandemic.

## Methods

### Design

All guidelines listed in the PRISMA (Preferred Reporting Items for Systematic Reviews and Meta-Analyses) statement were followed in performing this meta-analysis [30]. For this systematic review and meta-analysis, data were pooled from observational studies, including cohort, case-control, cross-sectional, and similar viable case studies. The study was registered on PROSPERO (International Prospective Register of Systematic Reviews; CRD42023410657).

### Search Strategy

We performed a simple search in the Google Scholar, PubMed, Scopus, Web of Science, and Embase databases to identify observational studies suitable for inclusion with the following search terms: “maternal anemia” OR “anemic condition” OR “poor hemoglobin levels” OR “pregnancy anemia” OR “anemia in pregnant women” OR “gestation anemia” AND “treatment” OR “intervention” OR “management” AND “effect” OR “effectiveness” AND “impact” OR “outcome.” Studies were restricted to those published in English from December 2019 to August 2022.

### Inclusion and Exclusion Criteria

The inclusion criteria for this study were as follows:

1. Studies that examined women of reproductive age who were part of any anemia prevention program or intervention, whether they were anemic or nonanemic according to World Health Organization criteria.
2. Observational, cross-sectional, prospective, or retrospective studies.
3. Studies that compared intervention approaches with control or comparator approaches.
4. Studies evaluating the effects of different interventions on pregnant women during the advent of the COVID-19 pandemic.

The exclusion criteria for this study were as follows:

1. Unrelated, duplicate, and missing information answering our research question.
2. Non–English-language studies.
3. Case reports/series.
4. Reviews.
5. Editorials.
6. Studies lacking a full text (unavailable or not yet published).
7. Articles without a DOI.
8. Studies with small sample sizes (<50 patients), due to low statistical power.

### Data Extraction

Both adjusted and nonadjusted data for pregnant women receiving interventions versus those in the comparator group were extracted to identify the most relevant confounding factors for subsequent pooling analysis. Two reviewers (JKM and DMF) scanned study titles and abstracts obtained from the initial database search and included relevant articles in a secondary pool. Next, two independent reviewers (FMW and KO) evaluated the full texts of these articles to determine if they met the study inclusion criteria. Any disputes were resolved through discussion and negotiation with a fourth reviewer (EMN). Only studies agreed upon by all reviewers were included in the final analysis.

The following data were obtained from all studies: title, first author, data collection year, region, sample size, study design, study setting (single or multicenter), intervention type, and the effect associated with each intervention approach. The analysis aimed to determine whether the intervention group was more likely to experience a better effect on maternal anemia mitigation, treatment, or management using end-result indicators such as hemoglobin levels. Further sensitivity and subgroup analyses were also conducted.

### Risk of Bias (Quality) Assessment

To assess the quality of randomized controlled trials (RCTs), the Cochrane Risk of Bias tool and Risk of Bias 2 tool [31] were used, evaluating domains such as randomization, deviations from intended interventions, missing outcome data, outcome measurement, and selection of reported results. For observational and cross-sectional studies, the National Institutes of Health tool was used [32]. Two to three reviewers independently assessed study quality, rating each of the 14 items as yes, no, or not applicable. Overall scores were calculated to classify studies as poor, fair, or good. To reduce bias, data checks were performed by reviewers who did not initially extract the data, though some overlap occurred in rare cases.

### Statistical Analyses

Review Manager 5.4.1 was used to calculate rate ratios (RRs) with 95% CIs, depicted using forest plots. Quantitative variables were summarized as total numbers and percentages. RRs that did not favor the intervention arm were noted. The effects on anemia prevention, control, management, and treatment were compared between intervention and control arms.

Heterogeneity was evaluated using the Cochran Q and Higgins tests, applying fixed-effects or random-effects models based on heterogeneity levels. Sensitivity adjustments were made to identify sources of heterogeneity by excluding studies one at a time. Subgroup analysis, cumulative analyses, and meta-regression were performed to test result consistency and the impact of confounders on anemia control. Publication bias was assessed using the Cochrane Risk of Bias tool.

## Results

### Included Articles and Quality Assessment

**Figure 1.**
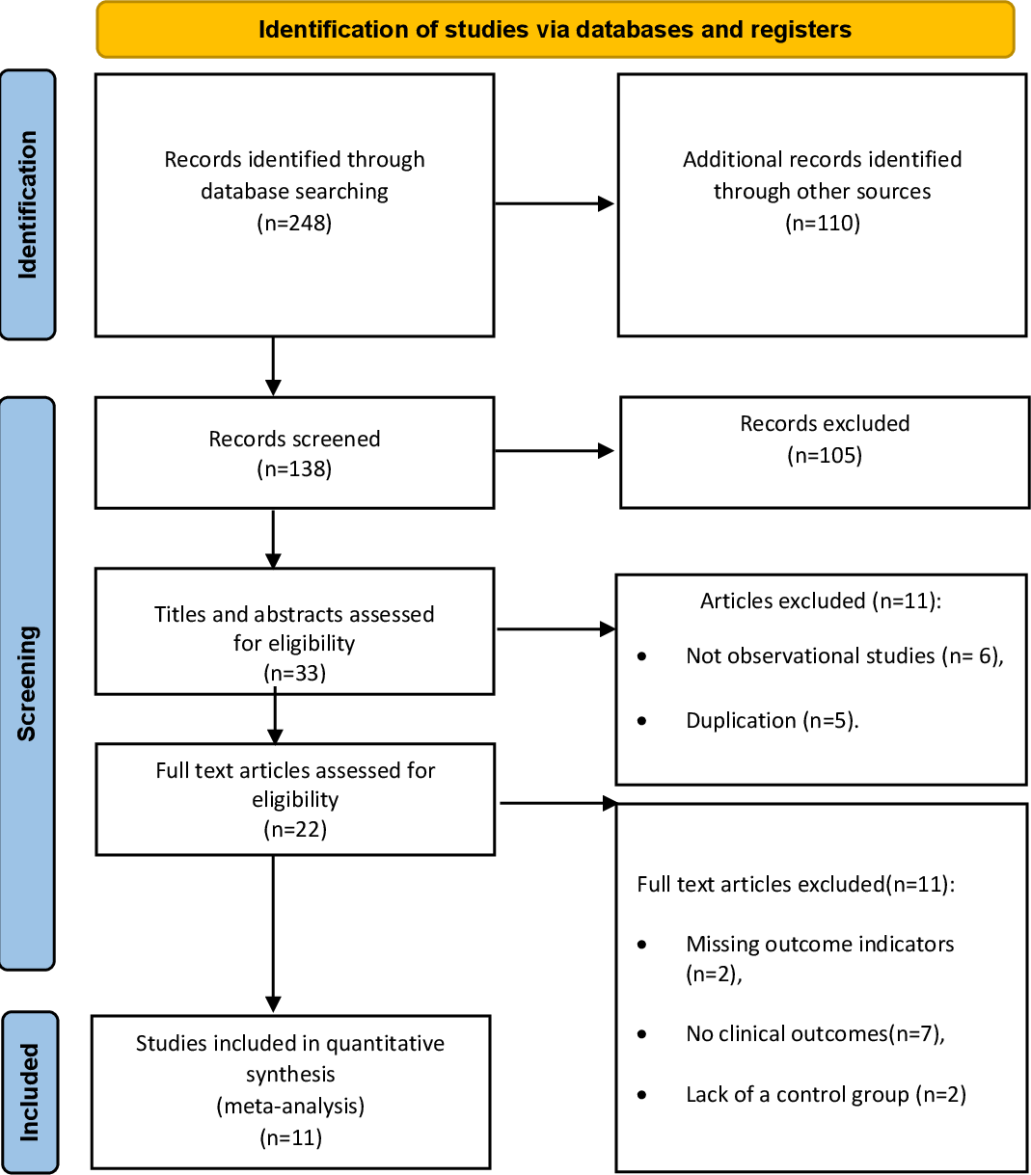
PRISMA (Preferred Reporting Items for Systematic Reviews and Meta-Analyses) flowchart of the study selection procedures.

The initial search of international databases using the specified keywords yielded 248 articles. After excluding 110 duplicates, 138 articles remained. Upon evaluating the titles and abstracts for appropriateness, 33 articles met the inclusion criteria. Additionally, 22 articles were excluded after full-text review for not meeting the inclusion criteria. Ultimately, 11 articles met the inclusion criteria [33-43] (Figure 1).

### Features of the Included Studies

The 11 included studies provided data for 6129 pregnant women in the advent of the COVID-19 pandemic [33-43]. Among the 6129 pregnant women included in the meta-analysis, 3591 (59%) were in the maternal anemia intervention group and 2538 (41%) were in the comparator group. The effects of the intervention were reported for 1921 participants (53.4%) in the intervention group and 1350 participants (53.1%) in the comparator group. The cumulative effect on maternal anemia for both groups ranged from 23% to 81%, with an average of 56%. The main outcome of this meta-analysis was the pooled effect of interventions on maternal anemia, assessed by increased hemoglobin levels and other parameters. The study designs included 4 RCTs (2 multicenter, 2 single-center), 3 cross-sectional studies (all multicenter), 2 prospective studies (1 multicenter, 1 single-center), 1 retrospective case-control study (single-center), and 1 quasi-experimental study (single-center). A summary of the studies is provided in Table S1 in Multimedia Appendix 1.

We evaluated the quality of observational studies using a modified Newcastle-Ottawa Scale, which includes 8 items across 3 subscales. Studies scoring ≥7 were considered high quality, though no universal standard exists. Out of 11 studies, the average score was 6.7, indicating moderate quality (score range: 5‐8; see Table S2 in Multimedia Appendix 1).

### The Pooled Effect of Interventions on the Prevention and Management of Maternal Anemia

**Figure 2.**
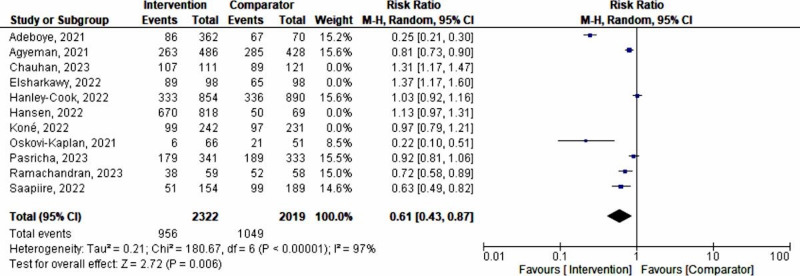
A forest plot of a meta-analysis of the effect of maternal anemia interventions [33-43]. M-H: Mantel-Haenszel.

**Figure 3.**
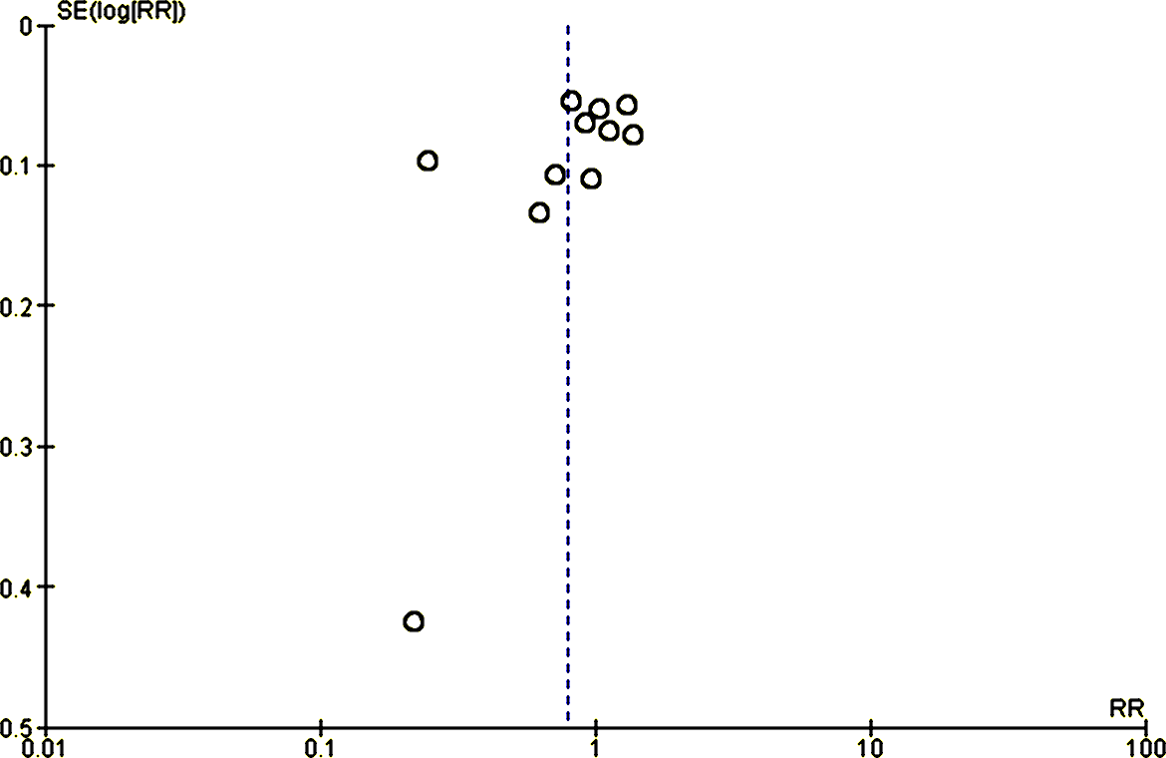
Funnel plot evaluating publication bias. RR: relative risk; SE: standard error.

The meta-analysis revealed a nonsignificant effect of the interventions on the prevention and management of maternal anemia as indicated by stabilized hemoglobin levels and other parameters (random-effects model RR 0.79, 95% CI 0.61‐1.02; *P*=.07; *χ*^2^_10_=286.98, *P*<.001; *I*²=97%). Based on the confidence interval, this indicated little knowledge about the effect and this imprecision affected the certainty in the evidence; thus, further information was needed before a more certain conclusion could be made (Figure 2). A funnel plot demonstrated an asymmetrical shape, depicting the presence of publication bias (Figure 3).

**Figure 4.**
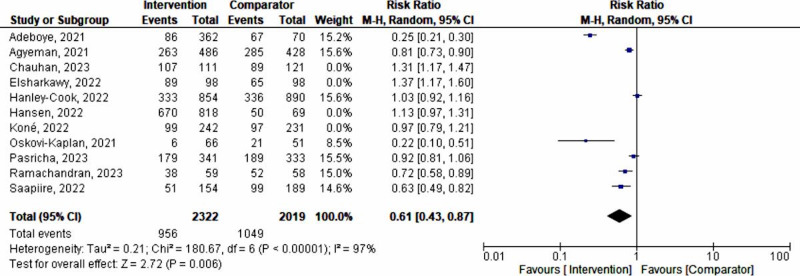
A forest plot of a meta-analysis on the effect of maternal anemia interventions after sensitivity analysis [33-43]. M-H: Mantel-Haenszel.

**Figure 5.**
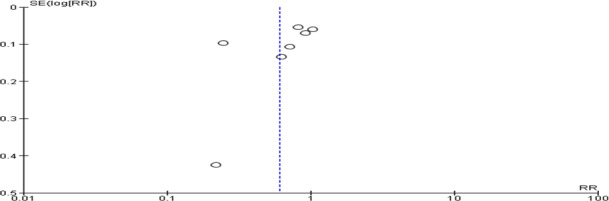
Funnel plot evaluating publication bias after sensitivity analysis. RR: rate ratio; SE: standard error.

A sensitivity analysis was performed to explore the impact of excluding or including studies in the meta-analysis based on sample size, methodological quality, and variance. After removing 4 studies (with 1788 pregnant women) [32-34,36] with wider 95% CIs, a total of 4341 pregnant women remained for analysis in the remaining studies [29-31,35,37-39], showing a shift in a random effects model (RR 0.61, 95% CI 0.43‐0.87; *P*=.006; *χ*²_6_=286.98, *P*<.001; *I*²=97%), revealing that the interventions had a 39% utility in preventing and managing maternal anemia during the advent of the COVID-19 pandemic (Figure 4). The funnel plot evaluating publication bias revealed considerable heterogeneity between all pooled studies for the updated analysis (*I*²=97%; *P*<.001; Figure 5).

### Subgroup Analysis and Investigation of Heterogeneity

Heterogeneity in the pooled effect estimates was considerably high for all 11 studies, with 1788 of 6129 (29%) evaluated subjects contributing to this variability. Therefore, it was necessary to perform subgroup analyses to identify possible variables or characteristics moderating the results.

Subgroup analysis with a random-effects model was conducted according to the type or form of the intervention used, including dietary iron supplementation (n=2176), education or dietary information (n=786), intravenous (IV) ferric carboxylmaltose (n=791), medicinal or herbal administration (n=914), IV sucrose (n=232), and other forms (n=1230). This analysis still showed considerable heterogeneity (*χ*²_₅_=38.92, *P*<.001; *I*²=87.2%).

The tests for the overall effect of dietary iron supplementation (*z*=0.92, *P*=.36), education or dietary information (*z*=.05, *P*=.96), ferric carboxylmaltose (*z*=1.01, *P*=.31), and other interventions (*z*=0.51; *P*=.61) all indicated no significant difference, with substantial heterogeneity (*I*²>90%).

**Figure 6.**
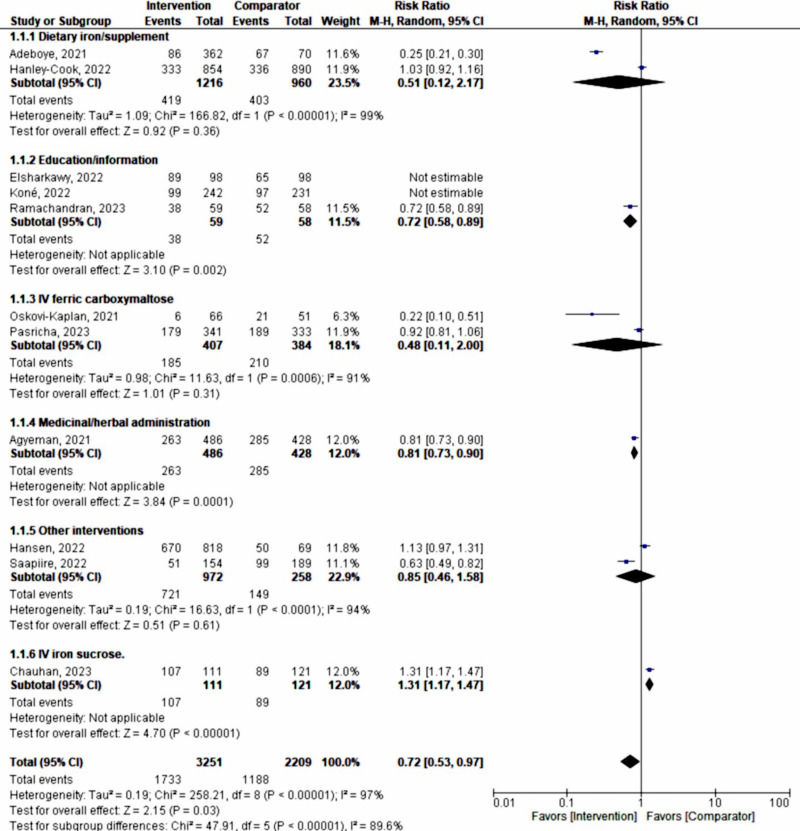
Subgroup analysis according to the type or form of intervention, showing similarly high heterogeneity as compared with the full meta-analysis [33-43]. IV: intravenous; M-H: Mantel-Haenszel.

**Figure 7.**
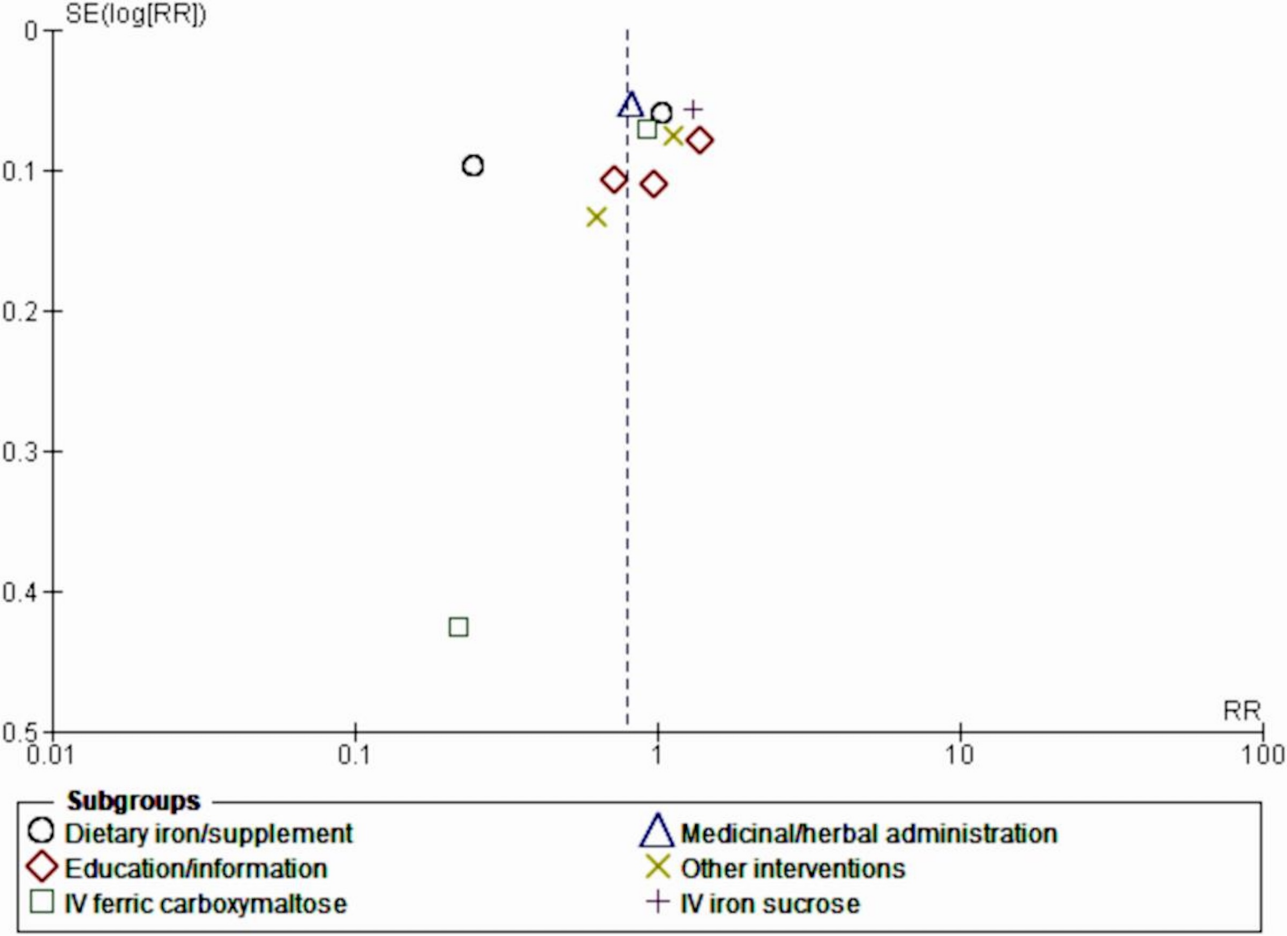
Funnel plot of the subgroup analysis (type or form of intervention). IV: intravenous; RR: rate ratio; SE: standard error.

Intravenous sucrose (RR 1.31, 95% CI 1.17-1.47; *z*=4.70; *P*<.001) demonstrated poor prevention and management of maternal anemia, favoring the comparator by 31%. Meanwhile, medicinal or herbal administration had a 19% effect on the prevention and management of maternal anemia (random-effects model RR 0.81, 95% CI 0.73-0.90; *P*=.006; Figure 6). Publication bias was further demonstrated by a funnel plot (Figure 7).

**Figure 8.**
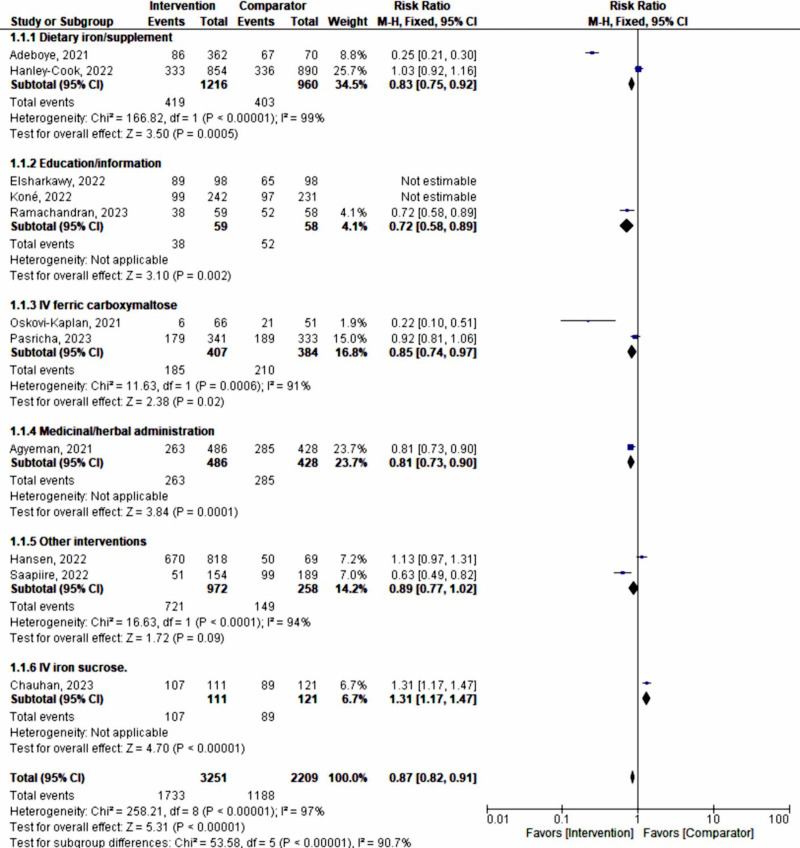
Subgroup analysis (fixed-effect model) according to the type or form of intervention [33-43]. M-H: Mantel-Haenszel.

Using a fixed-effect model, assuming one true effect size underlies each specific intervention form or approach, the subgroup analysis demonstrated the following significant influences on the prevention and management of maternal anemia: dietary iron supplementation (RR 0.83, 95% CI 0.75‐0.92; *P*<.001), IV ferric carboxylmaltose (RR 0.85, 95% CI 0.74‐0.97; *P*<.02), and medicinal or herbal administration (RR 0.81, 95% CI 0.73‐0.90; *P*<.001). However, all interventions still exhibited high heterogeneity (*I*²>90%; Figure 8).

The high heterogeneity obtained prompted a further sensitivity analysis on each subgroup to identify the group most strongly associated with heterogeneity. Following this analysis on subgroups (n=5228), by eliminating studies causing major heterogeneity [37,38,40], all intervention approaches against maternal anemia showed a pooled positive effect of 17% (fixed-effect model RR 0.83, 95% CI 0.79‐0.88; *P*<.001; *χ*²_₄_=2.93, *P*=.57; *I*²=0%).

**Figure 9.**
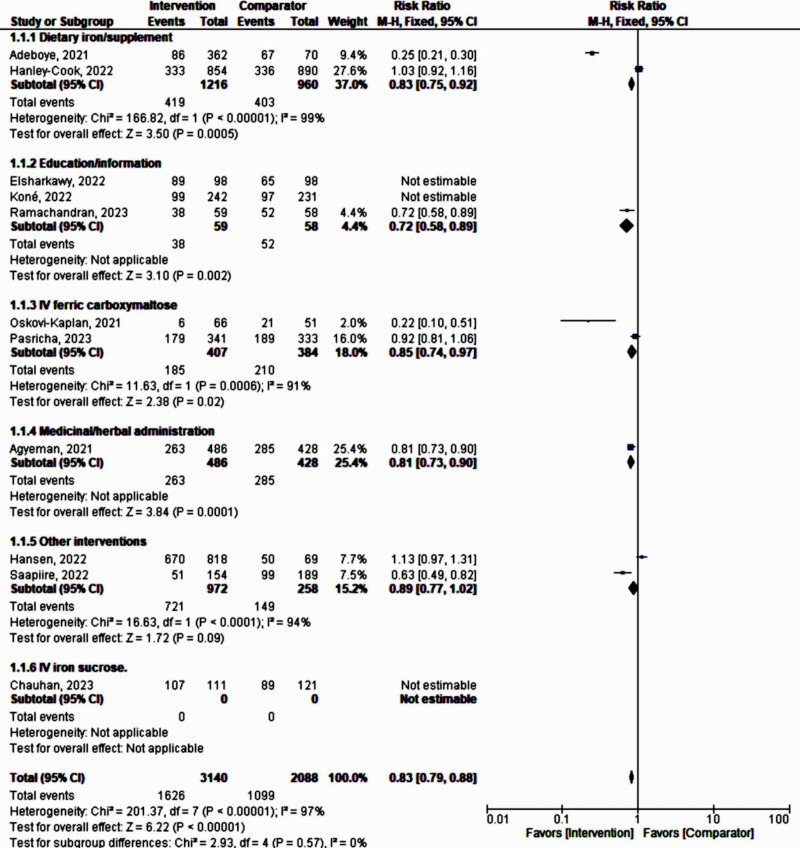
Sensitivity analysis on intervention type subgroups showing very low heterogeneity [33-43]. M-H: Mantel-Haenszel.

Education or information given to pregnant women (n=117) showed a 28% effect (RR 0.72, 95% CI 0.58‐0.89; *P*<.001). Medicinal or herbal administration had a 19% effect (RR 0.81, 95% CI 0.73‐0.90; *P*<.001; n=914). Dietary iron supplementation showed a 17% effect (RR 0.83, 95% CI 0.75‐0.92; *P*<.001; n=2176). IV ferric carboxylmaltose exhibited a 15% effect (RR 0.85, 95% CI 0.74‐0.97; *P*<.02; n=791; Figure 9).

**Figure 10.**
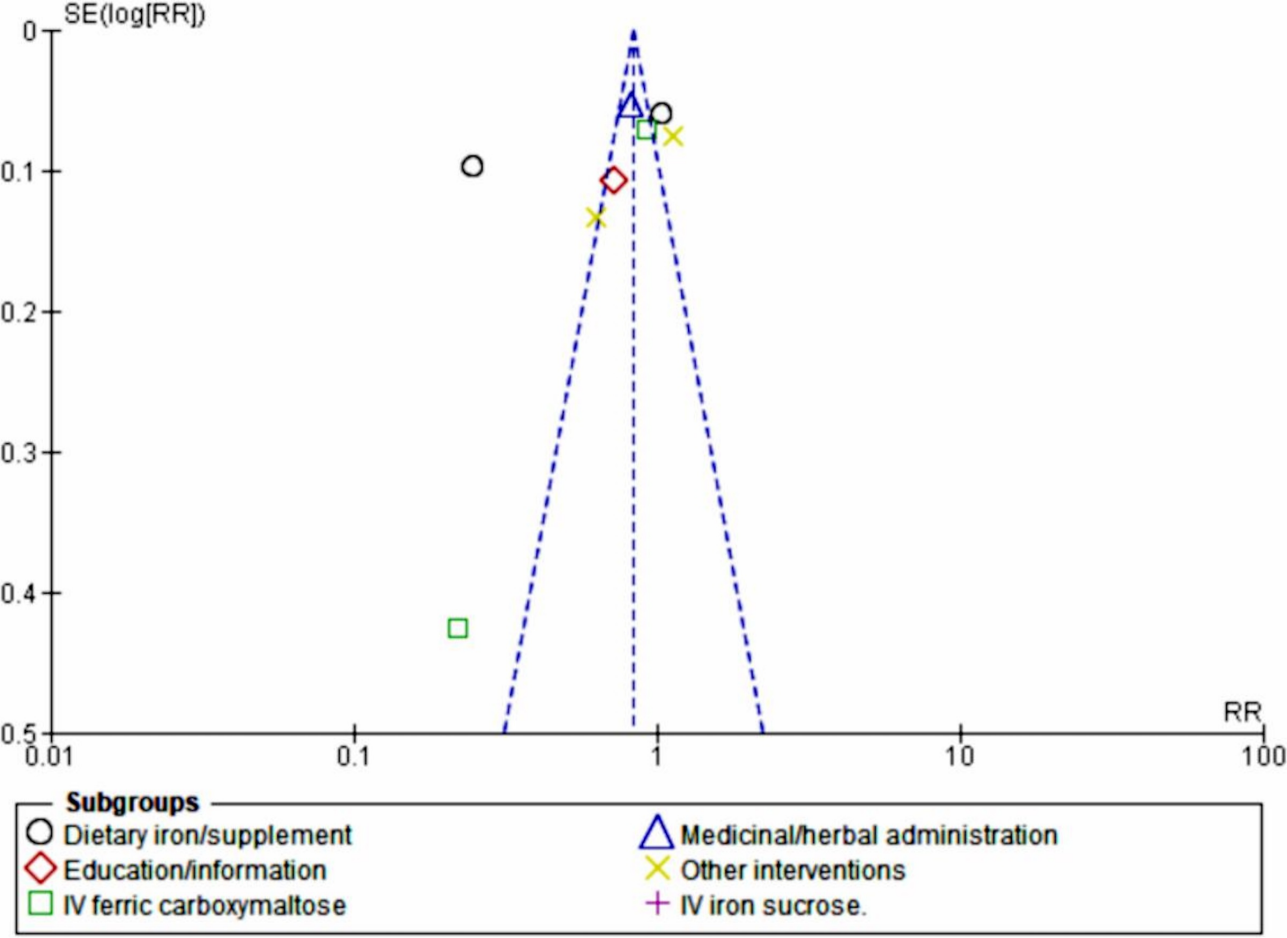
Funnel plot of the subgroup sensitivity analysis (type of intervention). IV: intravenous; RR: rate ratio; SE: standard error.

These findings were accompanied by greatly reduced publication bias and heterogeneity between the subgroups (*I*²=0%), as shown by the funnel plot (Figure 10).

### Subgroup and Sensitivity Analysis on the Possible Covariates

#### Location of the Pregnant Women

**Figure 11.**
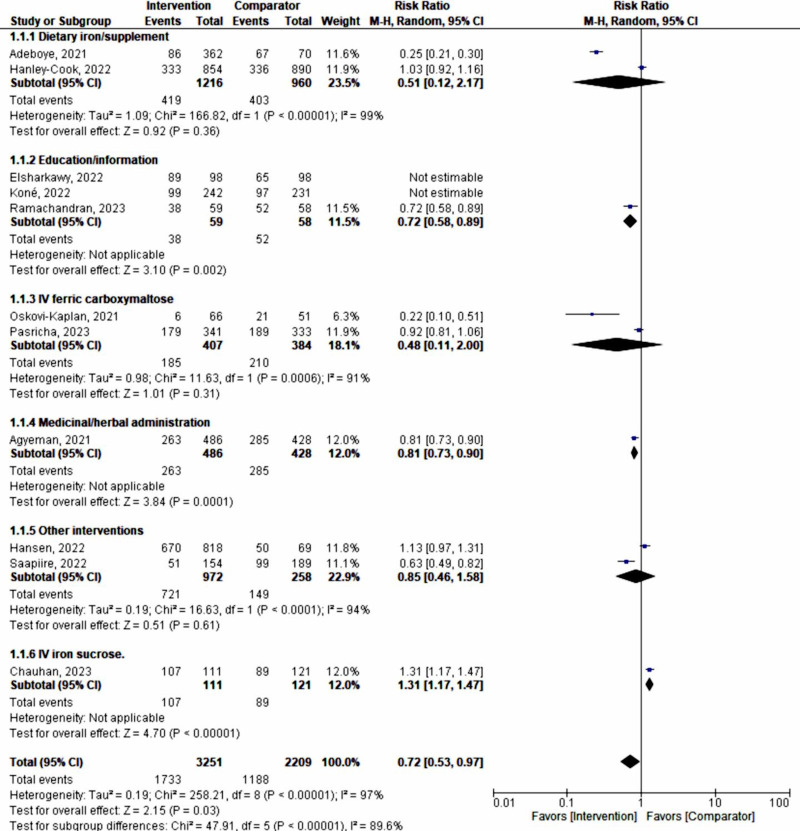
Subgroup analysis by the location of pregnant women during the advent of the COVID-19 pandemic [33-43]. M-H: Mantel-Haenszel.

Generally, maternal anemia interventions during the advent of the COVID-19 pandemic demonstrated a higher and significant effect (16%) in Africa (n=4580) compared to Asia and Europe (fixed-effects model RR 0.84, 95% CI 0.79‐0.89; *P*<.001; *χ*²_₅_=176.53, *P*<.001; *I*²=97%; Figure 11).

#### Study Setting

**Figure 12.**
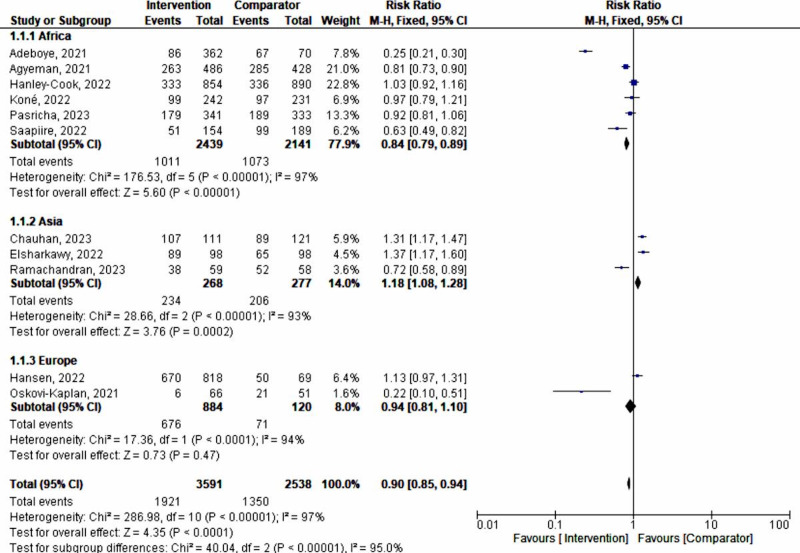
Subgroup analysis by study setting [33-43]. M-H: Mantel-Haenszel.

**Figure 13.**
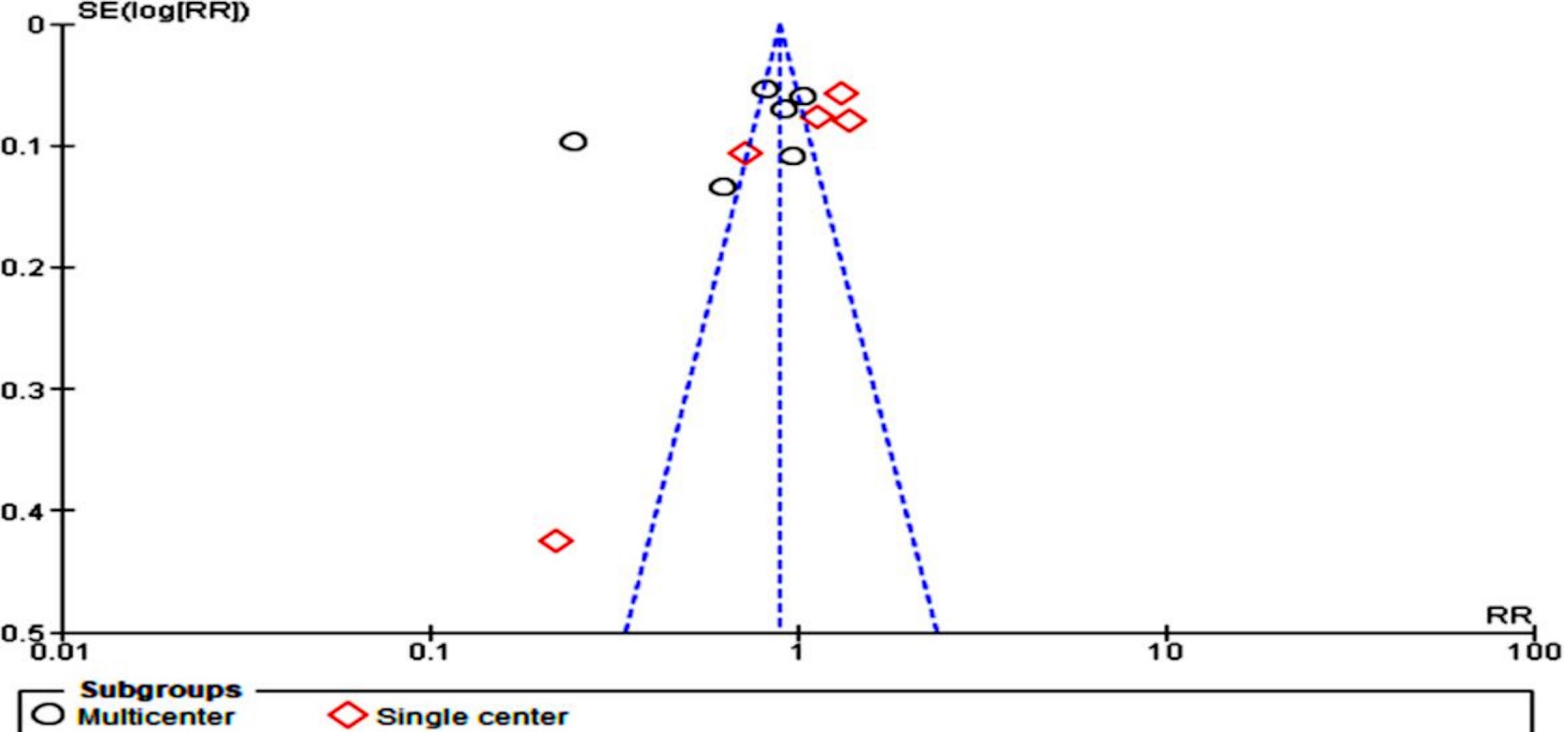
A funnel plot of subgroup analysis by study setting, showing that single-center studies (diamond shape) signified higher heterogeneity. RR: rate ratio; SE: standard error.

Similarly, multicenter studies (n=4580) showed a more significant predictive effect (16%) on maternal anemia intervention compared to single-center studies (n=1549; fixed-effects model RR 0.84, 95% CI 0.79‐0.89; *P*<.001; *χ*²_₅_=176.53, *P*<.001; *I*²=97%; Figure 12). The funnel plot demonstrated that most studies close to the mean effect were multicenter and associated with heterogeneity, with only one study tending to signify homogeneity (Figure 13).

#### Time or Year of Data Collection

**Figure 14.**
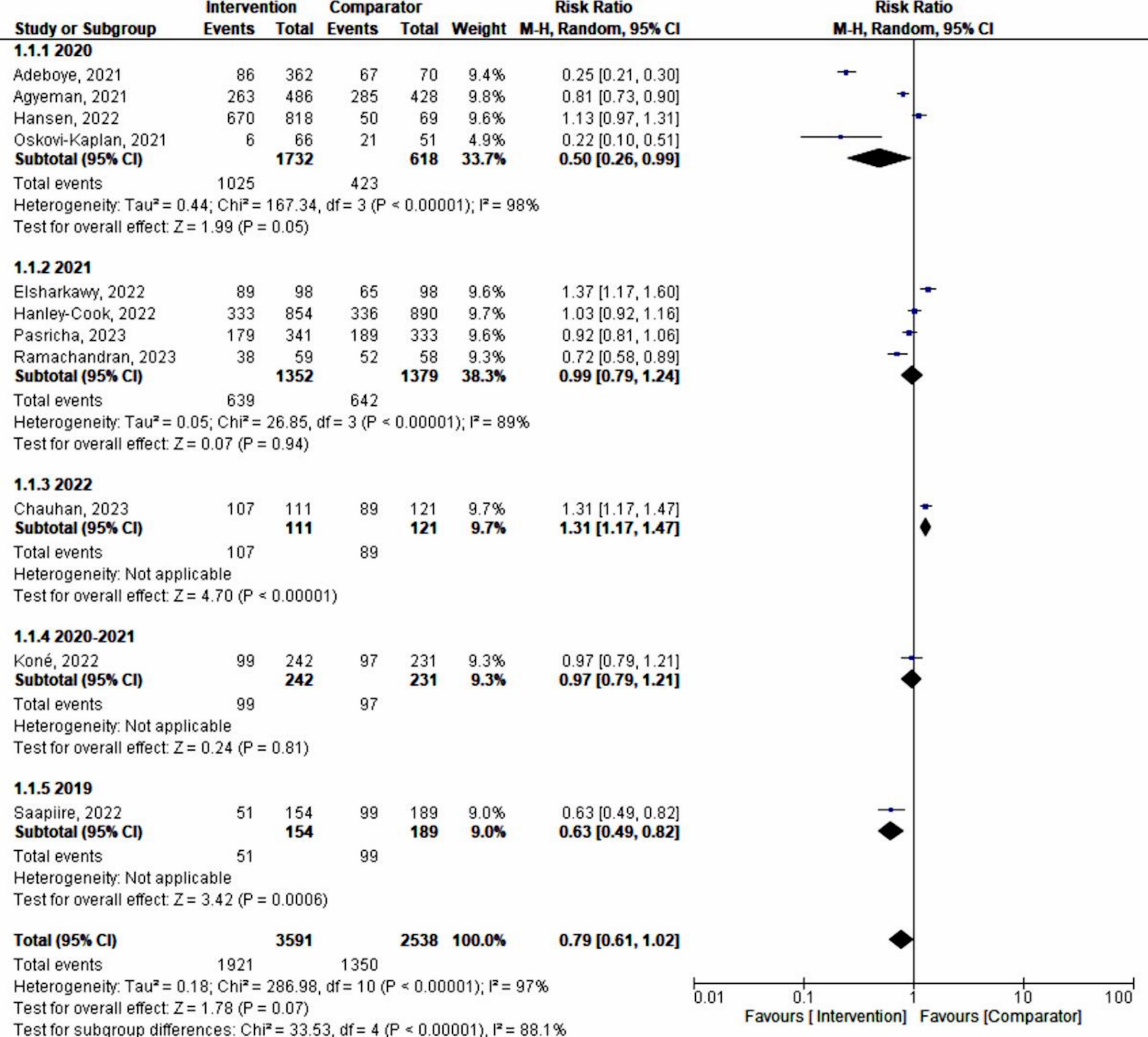
Subgroup analysis by the year the data were collected (random-effects model) [33-43]. M-H: Mantel-Haenszel.

**Figure 15.**
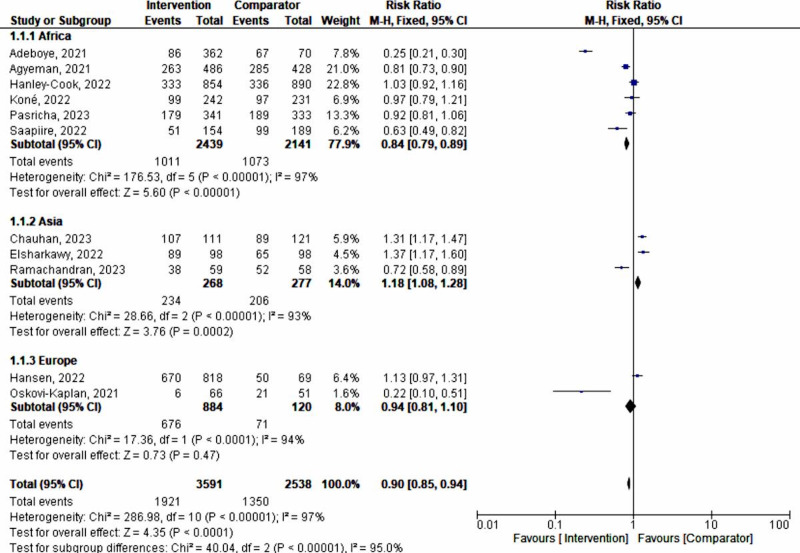
Subgroup analysis by the year the data were collected (fixed-effect model) 33-43. M-H: Mantel-Haenszel.

Studies whose data were collected in 2020, during the advent of the COVID-19 pandemic (n=2350), showed a significantly higher predictive effect (50%) on maternal anemia intervention compared to data from other times or years (random-effects model RR 0.50, 95% CI 0.26‐0.99; *P*<.05; *χ*²_₃_=167.34, *P*<.001; *I*²=98%; Figure 14). This finding was further supported by fixed-effect analysis, where the year 2020 showed a 28% effect (RR 0.72, 95% CI 0.67‐0.78; *P*<.001; *χ*²_₃_=167.34, *P*<.001; *I*²=98%; Figure 15).

### Statistical Justifications of the Use of Fixed-Effect or Random-Effects Models

The initial analysis used a random-effects model due to significant heterogeneity among the studies (*I*²=97%; *P*<.001), allowing for variations in true effect sizes across different populations and contexts. This model was suitable for handling diverse study designs, sample sizes, and intervention approaches. However, when assuming one true effect size for specific intervention forms, a fixed-effects model was used. This model was appropriate for evaluating interventions like dietary iron supplementation, IV ferric carboxylmaltose, and medicinal or herbal administration, despite high heterogeneity (*I*²>90%). By combining both models, the study leveraged the strengths of each approach, ensuring that the analysis accounted for both within-study and between-study variability. This dual approach enhanced the robustness and credibility of the findings, leading to more accurate and reliable pooled estimates for maternal anemia interventions.

## Discussion

### Principal Findings

This study found that while the overall effect of interventions on maternal anemia during COVID-19 was initially unclear due to high variability and publication bias, more focused analyses showed certain interventions—such as dietary iron supplementation, herbal/medicinal treatments, and IV ferric carboxylmaltose—were significantly beneficial. Sensitivity and subgroup analyses revealed these effects were strongest in studies conducted in Africa, studies conducted in multicenter settings, and especially among data collected in 2020, with a notable reduction in heterogeneity and a clearer positive impact on maternal hemoglobin levels.

The meta-analysis explored the effects of various interventions on maternal anemia among 6129 pregnant women during the COVID-19 pandemic. The primary outcome focused on the effectiveness of different interventions, such as dietary iron supplementation, education, intravenous iron therapy, and medicinal or herbal treatments. Although the overall quality of studies was moderate, the interventions showed varied effectiveness in managing and preventing maternal anemia. Sensitivity and subgroup analyses helped identify factors contributing to the observed heterogeneity in results. The interventions were generally more effective in Africa compared to Asia and Europe, and data collected during 2020 indicated a more significant impact. Overall, the study highlighted the need for further research to draw more definitive conclusions about the effectiveness of these interventions on maternal anemia.

This meta-analysis included 11 studies and revealed that the pooled intervention approaches had an effect on mitigating or reducing maternal anemia in the advent of the COVID-19 pandemic by 39%. Previous research has indicated a similar range of effect during the pandemic based on iron supplementation and iron and folic acid interventions (1.39, 0.33-2.45; *P*=.01 and 0.72, 0.36-1.07, *P*<.001, respectively) [15,27,44]. The general net cumulative effect of the interventions on maternal anemia ranged from 23% to 81% [6,45-47]. This is supported by recently published studies. Additionally, other meta-analyses have reported the outcomes of interventions on maternal anemia during the COVID-19 pandemic [28,44]. This analysis adds to the extensive consensus in the literature, which should motivate further research investigating the key aspects inherent to anemia control in pregnancy during similar pandemics.

Further, this systematic meta-analysis offers a more detailed view as it covers 11 studies from diverse regions capturing both single-center and multicenter studies. The heterogeneity was high even after subgroup analysis adjustments as per the specific cluster of intervention.

However, with fixed model analysis, dietary iron supplementation (17%), IV ferric carboxylmaltose (15%), and medicinal or herbal administration (19%) interventions significantly influenced the prevention and or management of maternal anemia. These findings are consistent with the guidance provided in the e-Library of Evidence for Nutrition Actions, where it is noted that daily iron and folic acid supplementation during pregnancy improve anemia [48], while the efficacy of intravenous ferric carboxylmaltose has been shown to be similarly positive for the condition [27,28,49-53]. In addition, past studies have also shown similar trends of significant control of maternal anemia through the use of medicinal or herbal treatments [29,54-56]. It is important to note that the effect found in this study as demonstrated by the pooled and specific interventions is seemingly lower as compared to the effects demonstrated by the earlier studies mentioned herein. Therefore, this can possibly be attributed to the influence of the COVID-19 pandemic in compromising the effectiveness of different anemia interventions.

The sensitivity analysis with the fixed-effect model on the subgroups by intervention type further showed a pooled positive effect of 17%. Notably, education or information interventions showed a 28% effect. Medicinal or herbal administration, iron supplementation, and IV ferric carboxylmaltose also had effects. The greatly reduced publication bias and heterogeneity between the subgroups following this sensitivity analysis provides evidence that using education and information to control maternal anemia is generally an efficient approach.

Anemia intervention in Africa generally had the highest effect as compared to other regions. However, this may not be due to best practices, as the prevalence of maternal anemia is higher in sub-Saharan Africa than in other regions [4,29,57-59]; instead, it may be due to more interventions being implemented to control anemia in Africa. Data collected from multicenter studies showed a more predictive effect (16%) of maternal anemia intervention as compared to single-center studies. Similar findings were reported by other similar reviews, although not during the COVID-19 pandemic [12,44,60]. In this context, the single-center studies had major heterogeneity as compared to multicenter studies.

Studies whose data were collected in the year 2020 in the advent of the COVID-19 pandemic had a more significant predictive effect (50%) on maternal anemia intervention as compared to other times or years of data collection. A subanalysis showed that the trend in maternal anemia effectiveness decreased with time from the year 2020 to 2021 and 2022. This fact is supported by a report asserting that the availability of nutritious foods in particular was affected by COVID-19 measures [7,61]. This was expected as nations concentrated on COVID-19 mitigation when it became a pandemic 2020 onward.

In addition, micronutrient intervention programs were affected during COVID-19, including disruptions of up to 75% for antenatal care programs in selected countries during the first months of the lockdown [29,62]. Furthermore, stockouts of iron and folic acid/multiple micronutrient supplementation may have occurred due to supply chain disruptions and programs no longer reporting stock information [9,63].

### Comparison to Prior Work

Prior studies have reported results that contrast with those presented here, with a better effect based on percentage reduction and/or hemoglobin mean standard deviation change on controlling maternal anemia [29,64-66]; however, these studies did not include data from the advent of the COVID-19 pandemic. In addition, a meta-analysis that targeted only RCTs [7,67] showed superiority in preventing anemia by the intervention as compared with the control. Moreover, a study focusing on hemoglobin mean level change demonstrated a similar trend in improving anemia control in pregnancy [9,68]. Of concern, as mentioned previously, most studies showed mixed outcomes relative to the outcome measure, with timelines of data collection in some being outside the scope of this study, which focused on the advent of the COVID-19 pandemic.

Similar findings were reported in a previous study, in which individual education through a pictorial handbook on anemia in conjunction with a counseling intervention program had a positive impact on hemoglobin and hematocrit levels for pregnant women with anemia in their third trimester of pregnancy [9,69]. The mean change in hemoglobin levels was also found to be significant in another study, which established that educational interventions can increase family support for maternal behaviors that can prevent anemia during pregnancy, such as improving adherence to taking iron supplements and maintaining a high intake of food containing iron [7,65]. Prior studies reported better outcomes from information package interventions compared to the current findings in the advent of the COVID-19 pandemic. Generally, an education package on maternal anemia control is part of an integrated approach where all the other intervention methods are included as part of the package [9,70]. This may be why the current findings show that this intervention had the highest effect on maternal anemia control.

The effects and impacts of specific disasters and/or calamities on maternal anemia interventions have been investigated previously. In one study, the COVID-19 crisis exacerbated maternal and child undernutrition and child mortality in low- and middle-income countries [28,71]. Further, measuring the effects of COVID-19 disruptions on the delivery of essential health and nutrition interventions has proven challenging, as resilient, real-time information systems were not well-established in many countries before the crisis [9,72]. A World Health Organization report surveyed the extent of disruptions across all health care services; such disruptions may have included disruptions to pregnancy anemia management and interventions [73]. Similarly, a study based in Africa found that health care services utilization in the advent of the COVID-19 pandemic was disrupted [29,74]. This could be expected to have affected and compromised standard interventions for mitigating maternal health issues. Another study demonstrated that the COVID-19 pandemic affected maternal health both directly and indirectly, including poor birth and maternal health outcomes [10,75]. This can explain the reduced effect of maternal anemia interventions.

Research shows that, in 2019, global anemia prevalence was 29.9% (95% uncertainty interval [UI] 27.0%-32.8%) in women of reproductive age, equivalent to over half a billion women aged 15‐49 years. Prevalence was 29.6% (95% UI 26.6%-32.5%) in nonpregnant women of reproductive age and 36.5% (95% UI 34.0%-39.1%) in pregnant women. Since 2000, the global prevalence of anemia in women of reproductive age has been stagnant, while the prevalence of anemia in pregnant women has decreased slightly [27,76]. Although more information is accumulating daily since the COVID-19 pandemic, subjective factors on pregnancy and the effect of the pandemic on health systems in African nations may have compromised the progress toward addressing anemia in general [29,77]. Given this, a few interlinked factors, including any similar pandemics, should be considered together as a single risk factor for maternal anemia.

### Strengths and Limitations

The study included 11 articles with 6129 participants and revealed a pooled intervention effect of 39% in preventing and managing maternal anemia. Interventions such as education (28%), medicinal administration (19%), iron supplementation (17%), and IV ferric carboxylmaltose (15%) showed substantial impact, especially in Africa. Multicenter studies were more predictive than single-center ones. Sensitivity analyses significantly reduced heterogeneity (*I*²=0%), increasing the reliability of results. Education and tailored strategies proved highly effective in low-resource settings and during crises, highlighting the importance of contextual interventions.

Several constraints may have affected the findings. First, most studies were retrospective, with only 4 RCTs contributing high-quality evidence. This could weaken the robustness of pooled estimates, though sensitivity analyses were conducted to mitigate this. Second, a lack of demographic details—such as participant age or gestational stage—led to inconsistent data and reduced comparability. Future studies should ensure thorough reporting to enhance clarity. Third, COVID-19 may have indirectly affected anemia metrics through influences on hemoglobin levels and nutritional access. These impacts underscore the need for pandemic-adjusted assessments in future meta-analyses. Publication bias may have exaggerated effectiveness, but comprehensive searches and statistical adjustments helped maintain validity.

### Future Directions

The effectiveness of maternal anemia interventions declined during the COVID-19 pandemic (2020‐2022), even for the most reliable approaches. Future pandemics call for rapid research into resilient solutions. Pregnant women should be screened for tailored interventions, and stakeholders must prioritize maternal health in emergency planning. Further studies should explore the mechanisms behind the reduced effectiveness and improve delivery systems.

This meta-analysis advances existing knowledge by using rigorous methodologies and expanded datasets from 11 articles with 6129 participants. It reveals novel insights, such as a 39% utility in preventing and managing maternal anemia, with significant impacts from education (28%), medicinal administration (19%), iron supplementation (17%), and IV ferric carboxylmaltose (15%). The study also highlights regional differences, particularly higher effectiveness in Africa, and underscores the importance of multicenter studies and ongoing research.

### Feasible Policy Recommendations

Based on this study, we make the following policy recommendations:

- Tailored regional interventions: focus on region-specific approaches, especially in Africa, to address unique challenges and maximize effectiveness.
- High-impact interventions: emphasize proven interventions like dietary iron supplementation, intravenous ferric carboxylmaltose, and medicinal or herbal administration for better prevention and management.
- Multicenter studies: encourage multicenter studies to improve the generalizability and reliability of results.
- Education and information: educate pregnant women about anemia prevention and management, providing dietary information and education.
- Time-specific considerations: tailor interventions to address unique circumstances, such as the advent of future pandemics, and resulting impacts on maternal anemia.

### Conclusion

The COVID-19 pandemic exposed critical gaps in maternal anemia management, underscoring the need for resilient health care strategies and enhanced data systems. Although meta-analytical evidence revealed modest yet significant intervention effects—especially from medicinal or herbal therapies, education, and dietary iron supplementation—these benefits were most evident in multicenter studies and African populations when high-heterogeneity outliers were excluded. This context-specific efficacy highlights the urgency for tailored approaches and further research to strengthen maternal and child health during future global crises, as emphasized [10,78].

## Supplementary material

10.2196/57626 Multimedia Appendix 1 Supplementary tables.

10.2196/57626 Checklist 1 PRISMA checklist.

## Abbreviations

IV: intravenous
PRISMA: Preferred Reporting Items for Systematic Reviews and Meta-Analyses
PROSPERO: International Prospective Register of Systematic Reviews
RCT: randomized controlled trial
RR: rate ratio
UI: uncertainty interval
