# Supplementary material for: Effects of Interventions for the Prevention and Management of Maternal Anemia in the Advent of the COVID-19 Pandemic: Systematic Review and Meta-Analysis
Source: JMIRx Med. 2025 Oct 6;6:e57626. doi: 10.2196/57626 (PMC12645416; doi:10.2196/57626)
Supplement: Multimedia Appendix 1 [file xmed-v6-e57626-s001.docx]

**Multimedia Appendix 1: Supplementary Tables.**

**Multimedia Appendix 1, Table S1.** Features of the Studies Included in the Meta-analysis (n = 11).

| **Study/Author** | **Location** | **Study Design & Setting** | **Intervention Approach** | **Year** | **Intervention Group** | **Comparator Group** | **Cumulative Effect (%)** |
| --- | --- | --- | --- | --- | --- | --- | --- |
| Adeboye et al., 2022 | Africa | gCS, bMC | Dietary iron intakes | 2020 | 86/363 | 67/70 | 35 |
| Agyeman et al., 2021 | Africa | aCS, bMC | SP dosage regimen (>3 doses under new policy) | 2020 | 263/486 | 285/428 | 60 |
| Chauhan et al., 2023 | Asia | cRCT, hSC | Intravenous iron sucrose | 2022 | 107/111 | 89/121 | 84 |
| Elsharkawy et al., 2022 | Asia | cRCT, hSC | Individual education via HIPP | 2021 | 89/98 | 65/98 | 79 |
| Hanley-Cook et al., 2022 | Africa | fP, bMC | IFA + fortified BEP supplement | 2021 | 333/854 | 336/890 | 38 |
| Hansen et al., 2022 | Europe | fP, gSC | Iron-deficiency anemia treatment | 2020 | 670/818 | 50/69 | 81 |
| Oskovi-Kaplan et al.,2021 | Asia | cRCC, hSC | Intravenous ferric carboxy-maltose | 2020 | 6/66 | 21/51 | 23 |
| Koné et al., 2023 | Africa | cRCT, bMC | Nutrition information | 2020–2021 | 99/242 | 97/231 | 41 |
| Pasricha et al., 2023 | Africa | cRCT, bMC | Ferric carboxy maltose | 2018–2021 | 179/341 | 189/333 | 38 |
| Ramachandran et al., 2023 | Asia | iQE, hSC | Individual nutrition education | 2021 | 38/59 | 52/58 | 77 |
| Saapiire et al., 2022 | Africa | hCS, bMC | ANC service utilization adequacy | 2019* | 51/154 | 99/189 | 62 |

*Abbreviations: aRCC = retrospective case-control; bMC = multicenter; cRCT = randomized clinical trial; dR = retrospective; eO = observational; fP = prospective; gCS = cross-sectional; hSC = single-center; iQE = quasi-experiment.*

| **Study** | **Case selection (maximum 4)** | **Comparability (maximum 2)** | **Exposure/outcome (maximum 3)** | **Total score** |
| --- | --- | --- | --- | --- |
| Adeboye et al., 2022 | 3 | 2 | 2 | 7 |
| Agyeman et al., 2021 | 3 | 2 | 1 | 6 |
| Chauhan et al., 2023 | 4 | 2 | 2 | 8 |
| Elsharkawy et al., 2022 | 3 | 1 | 2 | 6 |
| Hanley-Cook et al., 2022 | 4 | 1 | 2 | 7 |
| Hansen et al., 2022 | 3 | 2 | 3 | 8 |
| Oskovi-Kaplan et al.,2021 | 3 | 1 | 2 | 6 |
| Koné et al., 2023 | 4 | 1 | 2 | 7 |
| Pasricha et al., 2023 | 4 | 1 | 2 | 7 |
| Ramachandran et al., 2023 | 3 | 2 | 2 | 7 |
| Saapiire et al., 2022 | 3 | 1 | 2 | 6 |

**Multimedia Appendix 1, Table S2.** Newcastle-Ottawa scale for quality assessment and risk of bias.

**Mean score = 6.7**

The NOS evaluates studies across three domains:

- Selection (up to 4 stars)

- Comparability (up to 2 stars)

- Outcome or Exposure (up to 3 stars)

Each study can earn a maximum of 9 stars.

The mean Newcastle-Ottawa Scale (NOS) score across included observational studies was **6.7 out of 9**, indicating **moderately high methodological quality**. This reflects a generally acceptable level of rigor, with low to moderate risk of bias across selection, comparability, and outcome domains.
