# Supplementary material for: Effects of Interventions for the Prevention and Management of Maternal Anemia in the Advent of the COVID-19 Pandemic: Systematic Review and Meta-Analysis
Source: JMIRx Med. 2025 Oct 6;6:e57626. doi: 10.2196/57626 (PMC12645416; doi:10.2196/57626)
Supplement: Checklist 1 [file xmed-v6-e57626-s002.docx]

PRISMA 2020 Checklist – Filled

This checklist has been completed in accordance with the PRISMA 2020 guidelines.

| Section | Checklist Item | Page in Manuscript |
| --- | --- | --- |
| TITLE | Identify the report as a systematic review, meta-  analysis, or both. | Done, **Title** |
| ABSTRACT | Provide a structured summary including, as applicable: background; objectives; data sources; study eligibility criteria; participants and interventions; study appraisal and synthesis methods; results; limitations; conclusions and implications of key findings; systematic review  registration number. | Done, **Abstract** |
| INTRODUCTION - Rationale | Describe the rationale for the review in the context of  what is already known. | Done, **Introduction** |
| INTRODUCTION -  Objectives | Provide an explicit statement of the objective(s) or question(s)  the review addresses. | Done, **Objectives at Introduction** |
| METHODS - Eligibility criteria | Specify the inclusion and exclusion criteria for the review and how studies  were grouped for the syntheses. | Done, at **Methods** |
| METHODS - Information sources | Specify all databases, registers, websites, organizations, reference lists and other sources searched or consulted to identify studies. Specify the  date when each source was last searched or consulted. | Done, at **Methods** |
| METHODS - Search strategy | Present the full search strategies for all databases,  registers and websites, | Done, at **Methods** |

|  | including any filters and limits used. |  |
| --- | --- | --- |
| METHODS - Selection process | State the process for selecting studies (i.e., screening, eligibility, included in systematic review, and, if applicable,  included in the meta- analysis). | Done, at **Methods** |
| METHODS - Data collection process | Specify the methods used to collect data from reports, including how many reviewers collected data from each report, whether they worked independently, any processes for obtaining or confirming data from  study investigators. | Done, at **Methods** |
| METHODS - Data items | List and define all outcomes for which data were sought. Describe any assumptions  made about any missing or unclear information. | Done, at **Methods** |
| METHODS - Risk of bias | Specify the methods used to assess risk of bias in the  included studies. | Done, at **Methods** |
| METHODS - Effect measures | Specify for each outcome the effect measure(s) used in the synthesis or  presentation of results (e.g., risk ratio, mean difference). | Done, at **Methods**, under statistical analysis |
| METHODS - Synthesis methods | Describe the methods of handling data and combining results of studies, if done, including measures of consistency for  each meta-analysis. | Done, at **Methods**: **risk ratios (RRs),** random-effects model, fixed-effect model  - I² statistic, Cochran’s Q test, subgroup analyses and **meta-regression** |
| METHODS - Reporting bias assessment | Describe any methods used to assess risk of bias due to missing results in a  synthesis (arising from reporting biases). | Done, at **Methods**: Cochrane risk of bias tool, and the RoB 2 tool (7.0) |
| METHODS - Certainty assessment | Describe any methods used to assess certainty (or  confidence) in the body of evidence for an outcome. | Done, at **Methods**: Publication Bias: Cochrane Risk of Bias tool |
| RESULTS - Study selection | Give numbers of studies  screened, assessed for eligibility, and included in | Done, page 6: |

|  | the review, with reasons for  exclusions at each stage, ideally with a flow diagram. | Done, Flow Diagram |
| --- | --- | --- |
| RESULTS - Study characteristics | For each study, present characteristics for which data were extracted (e.g., study size, PICOS, follow-up  period) and provide the citations. | Done, Table 1 |
| RESULTS - Risk of bias in studies | Present assessments of risk of bias for each included  study. | Done, table 2-Newcastle-Ottawa scale for quality assessment and risk of bias. |
| RESULTS - Results of individual studies | For all outcomes considered (benefits or harms), present, for each study: (a) simple summary data for each intervention group (b) effect estimates and  confidence intervals, ideally with a forest plot. | Done, **at Results** , figure 1 |
| RESULTS - Results of syntheses | Present results of each meta-analysis done, including confidence  intervals and measures of consistency. | Done, **at Results** onwards |
| RESULTS - Reporting biases | Present assessments of risk of bias due to missing results (arising from reporting biases) for each  synthesis assessed. | Done, **at Results** onwards, via funnel plot publication bias |
| DISCUSSION | Provide a general interpretation of the results in the context of other  evidence, and implications for future research. | Done, **at Results** at Discussion section |
| OTHER INFORMATION -  Registration and protocol | Provide registration information for the review, including register name and registration number, and  indicate where the review protocol can be accessed. | registered (CRD-CRD42023410657). Protocol Accessible |
| OTHER INFORMATION -  Support | Describe sources of financial or non-financial support for the review, and the role of the funders or  sponsors in the review. | **At End Sections** |
| OTHER INFORMATION -  Competing interests | Declare any competing interests of review authors. | **At End Sections** |
